# Sex-based differences in outcomes of mitral valve surgery: A meta-analysis of propensity score-matched studies with reconstructed time-to-event data

**DOI:** 10.1016/j.ahjo.2025.100682

**Published:** 2025-11-21

**Authors:** Leo Consoli, Mir W. Majeed, Eren Cetinel, Pawel Lajczak, Ilias G. Koziakas, Hristo Kirov, Torsten Doenst, Tulio Caldonazo

**Affiliations:** a Federal University of Bahia, Salvador, Brazil; b Government Medical College, Srinagar, Jammu and Kashmir, India; c San Raffaele University, Milan, Italy; d Medical University of Silesia, Katowice, Poland; e Cardiac Surgery Department, Onassis Hospital, Athens, Greece; f Department of Cardiothoracic Surgery, Jena University Hospital, Friedrich Schiller University of Jena, Jena, Germany; g Department of Cardiothoracic Surgery, Weil Cornell Medicine, New York, United States of America

**Keywords:** Valvular heart disease, Cardiac surgery, Health equity, meta-analysis

## Abstract

**Background:**

Studies indicate worse outcomes for women undergoing mitral valve surgery, but this can be biased in the context of differences in risk profiles between sexes. We aimed to assess short- and long-term outcomes of mitral valve surgery in men and women using confounder-adjusted data.

**Methods:**

We searched PubMed, Embase, and the Cochrane Library for eligible propensity-score-matched studies. Analysis was performed for short-term (<30 days mortality and procedural complications) and long-term (>1 year mortality, reoperation, and mitral regurgitation) endpoints. A pairwise random-effects meta-analysis was done for short-term outcomes, pooling risk ratios (RR) with 95 % confidence intervals (CIs). A meta-analysis of Kaplan-Meier derived individual patient data was conducted for long-term endpoints. Cox frailty regression analysis was used to obtain hazard ratios (HR).

**Results:**

We included 12 studies (n = 55,616). No significant differences were observed in the short-term risks of death (RR 1.02; 95 % CI: 0.91–1.15; p = 0.72), stroke (RR 1.04; 95 % CI: 0.87–1.26; p = 0.65), kidney injury (RR 0.97; 95 % CI: 0.71–1.32; p = 0.85), atrial fibrillation (RR 0.96; 95 % CI: 0.81–1.14; p = 0.61), or pacemaker implantation (RR 0.93; 95 % CI: 0.84–1.02; p = 0.1). The hazards of long-term mortality (HR 0.97; 95 % CI: 0.91–1.03; p = 0.3) and reoperation (HR 1.65; 95 % CI: 0.39–6.91; p = 0.5) were similar between sexes. However, women had a higher hazard of recurrent mitral regurgitation (HR 1.61; 95 % CI: 1.08–2.37; p = 0.018).

**Conclusions:**

This meta-analysis found no sex-based differences in short- or long-term mortality, reoperation rates, or procedural complications following mitral valve surgery. A higher hazard of recurrent mitral regurgitation was observed in women.

## Highlights

• Meta-analysis of 12 propensity score-matched studies comparing mitral valve surgery based on sex.
• No significant difference in short-term mortality or in adverse procedural complications.
• No significant sex-based difference in long-term mortality or reoperation.
• A higher hazard for long-term recurrent mitral regurgitation was seen in women.

## Graphical abstract

Study design and findings at a glance.

**Figure:**
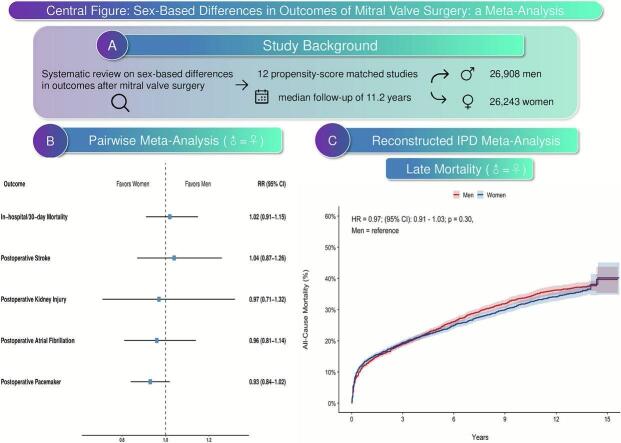

## 1 Introduction

Mitral valve surgery (MVS) is the standard treatment for patients with severe symptomatic mitral valve disease or hemodynamic impairment, performed either through mitral valve repair (MVr) or mitral valve replacement (MVR) [1]. Modern outcomes of MVS are excellent, with low mortality, durable efficacy, and infrequent reoperations [2]. However, a persistent concern in cardiac surgery is the presence of sex-based disparities in the outcomes of structural cardiac procedures, including MVS.

Several studies have reported worse outcomes for women following MVS, including higher in-hospital and long-term mortality, lower rates of MVr, more frequent recurrent regurgitation, and fewer concomitant procedures [3,4]. Women presenting for valve surgery are typically older, with a higher comorbidity burden and more advanced disease, which may lead to different surgical treatments and contribute to the observed disparities [3]. In this context, matching patients based on baseline characteristics, procedural risk, and disease severity enables less biased comparisons.

To address this lingering question in the literature, we conducted a systematic review and meta-analysis of propensity score-matched studies to evaluate sex-related differences in short- and long-term outcomes following MVS.

## 2 Methods

This systematic review and meta-analysis was conducted in accordance with the Cochrane Collaboration Handbook for Systematic Reviews of Interventions and adheres to the Preferred Reporting Items for Systematic Reviews and Meta-Analyses (PRISMA) guidelines [5,6]. The study protocol was prospectively registered in the International Prospective Register of Systematic Reviews (PROSPERO; number CRD420251012471). A PRISMA checklist was completed and can be found in Supplementary Appendix 1. As this is a systematic review and meta-analysis of secondary, de-identified data, patient consent and institutional review board approval was not necessary.

### 2.1 Eligibility criteria

The population of interest comprised patients undergoing MVS for mitral valve stenosis or regurgitation due to any etiology. We considered “women” as the intervention (exposure) group and “men” as the control. We included studies that reported at least one mortality outcome, either short- or long-term. To minimize bias due to confounding, we limited inclusion to studies that adjusted for baseline characteristics by propensity score-matching or related methods (e.g., inverse probability treatment weighting). To reduce the risk of publication bias, conference abstracts were also eligible for inclusion. No language restrictions were applied.

### 2.2 Search strategy and data collection

We performed a literature search on PubMed, Embase, and the Cochrane Library using the search strategy detailed in Supplementary Appendix 2. Studies were searched starting from March 2013 up to March 2025. The study selection process was independently performed by three reviewers (L.C, E.C and I.K.). Disagreements were resolved by consensus. After removing duplicates, the authors initially screened titles and abstracts and then assessed full-text articles for inclusion. We also manually screened the references of included studies for potential records. Data extraction was conducted using a standardized form by L.C. and E.C., and confirmed by M.W.M. We collected information on sample size, study design, patient characteristics, and clinical outcomes. Corresponding authors were contacted by email in cases of missing data.

### 2.3 Outcomes of interest

Short-term outcomes were defined as events occurring during the hospital stay or within 30 days postprocedure. These included early mortality, stroke, acute kidney injury, postoperative atrial fibrillation, and permanent pacemaker implantation. Long-term outcomes were those assessed after a minimum follow-up period of 1-year and included all-cause mortality, mitral reoperation, and recurrent mitral regurgitation. Outcome definitions can be found in Supplementary Appendix 3, Supplementary Table S1.

### 2.4 Risk of bias assessment

Risk of bias assessment for the included studies was performed independently by L.C. and I.K. using the Cochrane tool for assessing risk of bias in non-randomized studies (ROBINS-I) [7]. Studies were categorized as having low, moderate, or serious risk of bias based on the following domains: confounding; selection of participants; classification of interventions; deviations from intended interventions; missing data; measurement of outcomes; and selection of the reported result. Disagreements were resolved through consensus.

### 2.5 Statistical analysis

A pairwise meta-analysis was performed for short-term endpoints using risk ratio (RR) with 95 % confidence intervals (CIs). A two-tailed p-value of less than 0.05 was considered statistically significant. We used the Inverse Variance method with a random-effects model. Statistical heterogeneity among studies was assessed using Cochran's Q test, Tau^2^, and I^2^ statistics with DerSimonian and Laird's method. We classified heterogeneity as unimportant (I^2^ < 25 %), moderate (25 % < I^2^ < 50 %), substantial (50 < I^2^ < 75 %), and considerable (I^2^ > 75 %). Publication bias was evaluated using funnel plots and Egger's regression test for funnel plot asymmetry. Patient and procedural characteristics were pooled as means and compared using mean difference.

A meta-analysis of Kaplan-Meier–derived individual patient data was performed for long-term endpoints using the IPDfromKM method [8]. The proportional hazards assumption was assessed visually and tested using Schoenfeld residual plots and Grambsch−Therneau test. A Cox frailty regression model was used to obtain hazard ratios (HRs) and their 95 % CIs. Study groups were considered as a fixed effect (men vs women) and between-study heterogeneity was assessed by inclusion of a “study” frailty term, where individual studies were modeled as a random effect using random intercepts. If the proportional hazards assumption was violated, the difference in restricted mean survival time (dRMST) was also calculated from each individual patient dataset and pooled in a random-effects meta-analysis, following prior recommendations as the most accurate approach [9]. Time-based HR and dRMST were modeled using splines. A mixed-effects meta-regression was performed for short- and long-term mortality using important covariates to assess for moderators. A subgroup analysis was conducted comparing each surgical approach (MVr vs MVR). We used RStudio, version 4.2.2 (R Foundation for Statistical Computing). A complete description of the statistical methods is laid out in Supplementary Appendix 4.

## 3 Results

### 3.1 Study selection and baseline characteristics

**Fig. 1:**
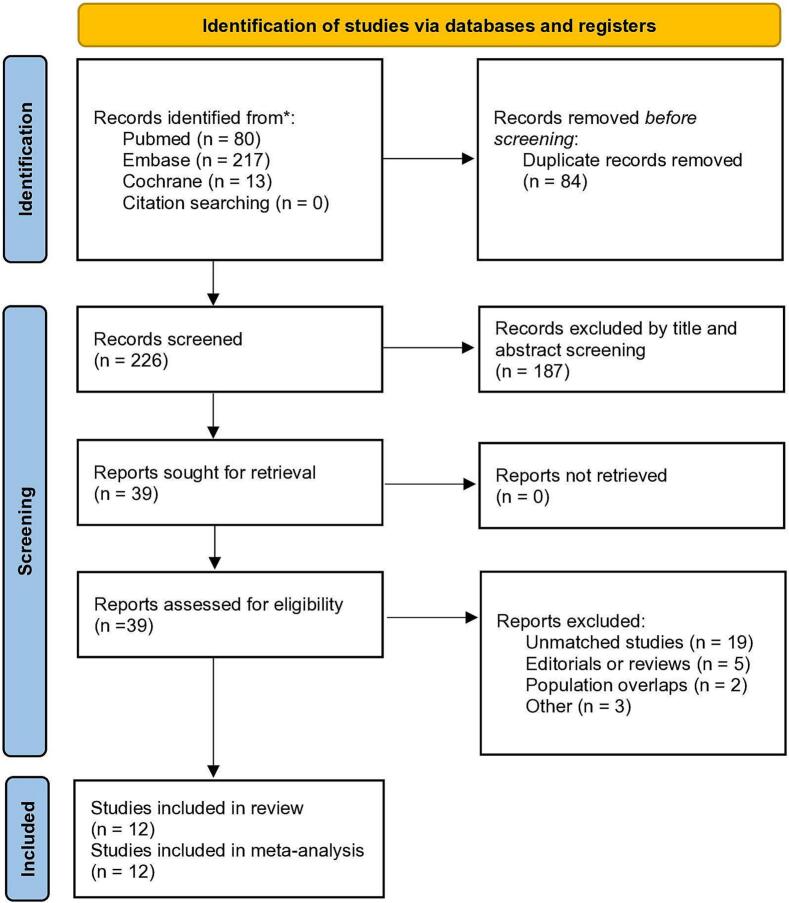
PRISMA flowchart for the systematic review. Sequential steps of study search and triage from databases with reasons for exclusion.

The search strategy identified 310 results (Fig. 1). After the removal of duplicate records and exclusion based on title and abstract screening, 39 studies remained for full-text review according to the inclusion and exclusion criteria. Of these, twelve studies met all the inclusion criteria [[10], [11], [12], [13], [14], [15], [16], [17], [18], [19], [20], [21]]. Excluded fully read articles with justifications can be found in Supplementary Appendix 5, Supplementary Table S2.

The analysis included a population of 55,616 patients with a mean follow-up of 11.3 years (Table 1). All included studies were retrospective cohorts and published from 2019 to 2025. Ten studies reported short-term mortality and seven reported short-term adverse events. Eleven studies reported long-term mortality, but only three reported long-term reoperation and recurrent regurgitation. One study was only available as a conference abstract and only contributed to the subgroup analysis [21]. Four studies presented isolated analysis for MVr and three for MVR. Baseline patient and operative characteristics are described in Table 2. Importantly, there was probable overlap between the studies by Kandula et al. and Kislitsina et al. [14,15]. As such, we only pooled these studies for different outcomes.

**Table 1:** Baseline characteristics of included studies.

Study; year	Country; period	Surgery	Adjustment method	Included population	Sample size	Follow-up, (years)
Chang; 2024 [10]	Taiwan; 2000–2018	MVr/MVR	PSM	MS/MR	12,724	14
El Andari; 2020 [11]	Canada; 2004–2018	MVR	PSM	MS/MR	622	16
El Andari; 2021 [12]	Canada; 2004–2018	MVr	PSM	Severe MS/MR	376	16
Munoz-Rivas; 2020 [13]	Spain; 2001–2015	MVR	PSM	Severe MR	34,712	In-hospital
Kandula; 2021 [14]	USA; 2004–2017	MVr/MVR	PSM	Degenerative MR	540	10
Kislitsina; 2019 [15]	USA; 2004–2017	MVr/MVR	PSM	MS/MR	846	12
Liu; 2023 [16]	China; 2010–2019	MVr	PSM	Degenerative MR	662	5.1
Malik; 2024 [17]	Canada; 2008–2023	MVr	IPTW	Degenerative MR	490	5.63
Passos; 2023 [18]	Switzerland/Germany; 2013–2022	MVr/MVR	PSM	MS/MR	202	8
Saijo; 2024 [19]	USA; 2010–2020	MVr/MVR	PSM	Severe calcific MS	64	14.67
Long; 2025 [20]	United Kingdom; 2017–2018	MVr/MVR	PSM	MR	76	8
Bernard; 2023^&^ [21]	Canada; 2002–2019	MVr/MVR	IPTW	Degenerative MR	1804	16

**&**: Abstract only; **IPTW:** inverse probability of treatment weighting; **MVr:** mitral valve repair; **MVR:** mitral valve replacement; **MR:** mitral regurgitation; **MS:** mitral stenosis; **NA:** not available; **PSM:** propensity score matching; **USA:** United States of America.

**Table 2:** Patient and procedural characteristics.

Variables	Overall (n = 55,616)	Men (n = 26,908)	Women (n = 26,243)	MD (95 % CI)	p-value
Age, in years	63.40 (61.69–65.11)	63.11 (60.81–65.40)	63.69 (61.15–66.23)	0.58 (−2.76–3.92)	0.733
MVr, in %	29.80 (29.06–30.05)	35.67 (34.58–36.78)	23.79 (22.79–24.82)	−11.88 (−13.56 to −10.20)	**<0.001**
LVEF, in %	60.62 (58.95–62.30)	60.23 (57.81–62.65)	61.03 (58.71–63.34)	0.8 (−2.53–4.13)	0.638
Hypertension, in %	50.9 (50.11–51.68)	51.13 (50.02–52.24)	50.66 (49.55–51.77)	−0.47 (−2.06–1.12)	0.56
Diabetes mellitus, in %	16.26 (15.93–16.59)	16.81 (16.34–17.28)	15.7 (15.24–16.17)	−1.11 (−1.76 to −0.46)	**<0.001**
Atrial fibrillation, in %	47.71 (47.27–48.15)	46.23 (45.61–46.85)	49.23 (48.61–49.86)	3 (2.13–3.87)	**<0.001**
CAD, in %	27.96 (27.56–28.37)	30.74 (30.16–31.32)	25.11 (24.55–25.68)	−5.63 (−6.45 to −4.81)	**<0.001**
BMI, in kg/m^2^	26.40 (25.57–27.24)	26.40 (25.3–27.5)	26.41 (25.14–27.67)	0.01 (−1.55–1.57)	0.992
CPB time, in minutes	126.23 (110.23–142.23)	129.64 (106.69–152.58)	122.74 (100.45–145.03)	−6.9 (−39.11–25.31)	0.672
ACC time, in minutes	94.49 (83.82–105.15)	96.91 (81.51–112.3)	92 (77.24–106.75)	−4.91 (−26.04–16.22)	0.65
10-year mortality, in %	32 (33–31)	32 (33–31)	32 (33–30)	0 (−2.05–2.05)	1
10-year reoperation, in %	1 (0.22–1.78)	1 (0–1)	1 (0–3)	0 (−1.22–1.22)	1
10-year recurrent MR, in %	12.96 (10.27–15.65)	10 (7–13)	16 (12–21)	6 (0.59–11.41)	**0.03**

Pooled means with 95 % confidence intervals (CI). **ACC:** aortic cross-clamp; **BMI:** body mass index; **CAD:** coronary artery disease; **CPB:** cardiopulmonary bypass; **LVEF:** left ventricular ejection fraction; **MD:** mean difference; **MVr:** mitral valve repair; **MR:** mitral regurgitation.

Statistically significant p-values are highlighted in bold.

In the overall population, women received less MVr (23.8 % vs 35.7 %), had more atrial fibrillation (49 % vs 46 %) and less coronary artery disease (25 % vs 30.5 %) and diabetes mellitus (15.7 % vs 16.8 %). The baseline characteristics of individual studies are found in Supplementary Table S3.

### 3.2 Short-term outcomes

**Fig. 2:**
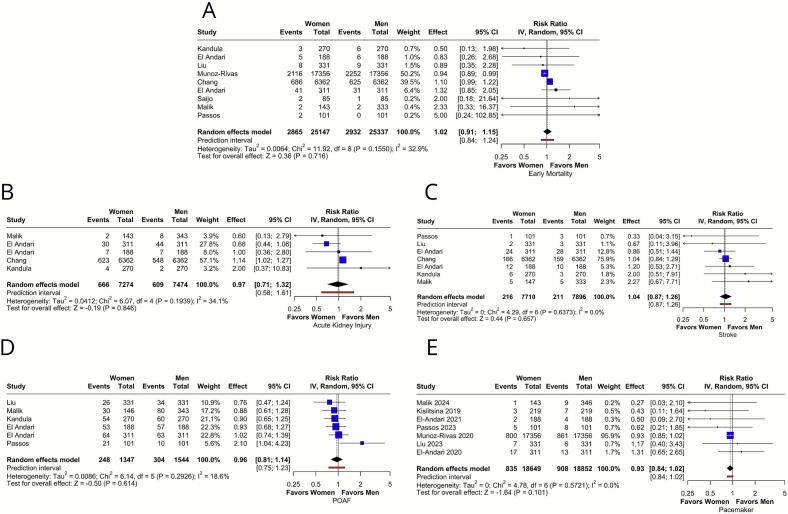
Forest plots of the short-term outcomes. **(A):** Forest plot for short-term mortality showing comparable risks among groups; **(B):** Forest plot for kidney injury showing comparable risks among groups; **(C):** Forest plot for stroke showing comparable risks among groups; **(D):** Forest plot for postoperative atrial fibrillation showing comparable risks among groups; and **(E):** Forest plot for pacemaker implantation showing comparable risks among groups. **CI:** confidence interval; **POAF:** postoperative atrial fibrillation; **RR:** risk ratio.

There was no significant difference in short-term mortality (RR 1.02; [95 % CI: 0.91–1.15]; p = 0.72; I^2^ = 32.9 %; Fig. 2A), with moderate heterogeneity. Some asymmetry was observed in the funnel plot (Supplementary Fig. S1), but it was not confirmed by Egger's test (p = 0.29). In a leave-one-out analysis, men or women could be statistically favored depending on the excluded study, indicating a lack of robustness (Supplementary Fig. S2). Regarding kidney injury, no significant sex-based differences were observed (RR 0.97; [95 % CI: 0.71–1.32]; p = 0.85; I^2^ = 34.1 %; Fig. 2B), with moderate heterogeneity. Comparable outcomes were also found for the risks of postoperative stroke (RR 1.04; [95 % CI: 0.87–1.26]; p = 0.66; I^2^ = 0.0 %; Fig. 2C), postoperative atrial fibrillation (RR 0.96; [95 % CI: 0.81–1.14]; p = 0.61; I^2^ = 18.6 %; Fig. 2D), and need for pacemaker implantation (RR 0.93; [95 % CI: 0.84–1.02]; p = 0.10; I^2^ = 0.0 %; Fig. 2E), with non-significant heterogeneity. Leave-one-out analyses were unremarkable, while some asymmetry was seen in the funnel plot for pacemaker implantation (Supplementary Figs. S3–S7).

### 3.3 Long-term outcomes

**Fig. 3:**
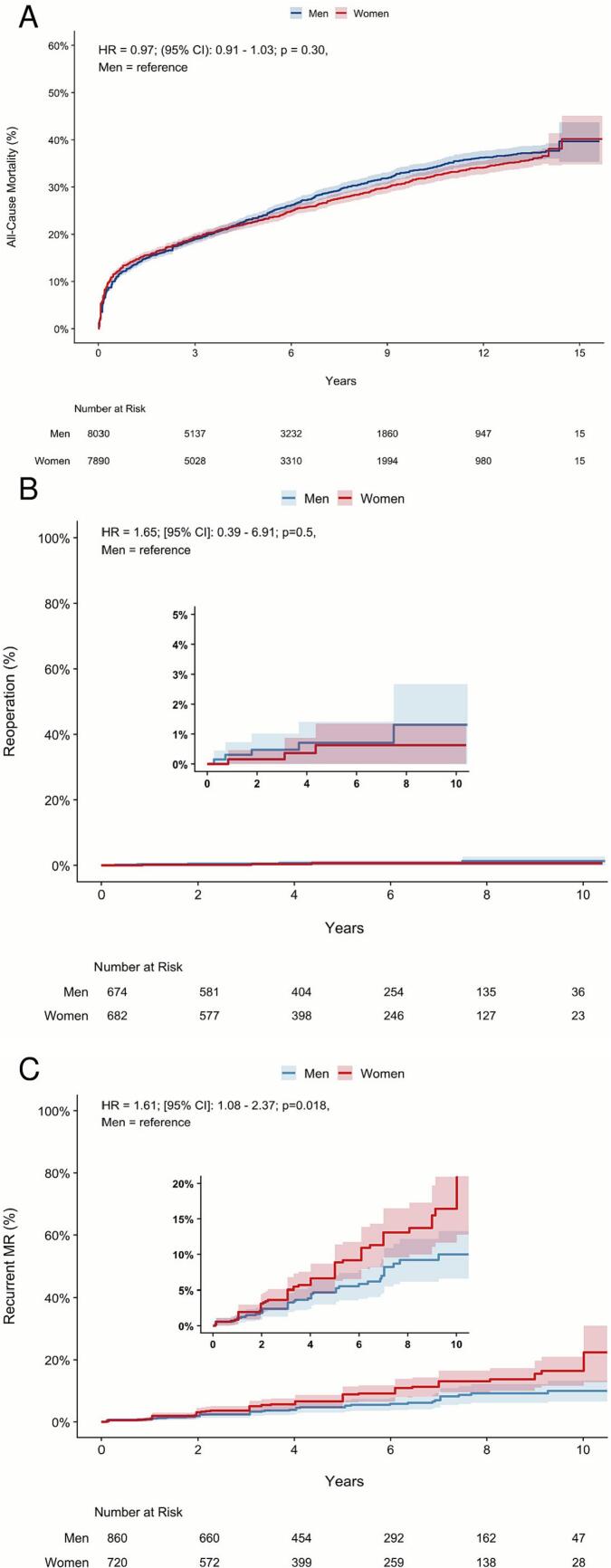
Kaplan-Meier curves of the long-term outcomes. **(A)**: Curve for long-term all-cause mortality showing comparable hazards among groups; **(B)**: Curve for long-term reoperation showing comparable hazards among groups; **(C)**: Curve for long-term recurrent mitral regurgitation showing a higher hazard in women. **CI:** confidence interval; **HR:** hazard ratio.

Over a 16-year period with 88,017 patient-years, no significant difference in mortality was found (HR = 0.97; [95 % CI]: 0.91–1.03; p = 0.30; Fig. 3A). Survival at five, ten and fifteen years was 77, 68 and 60 % for women and 76, 66 and 60 % for men. The proportional hazards assumption was violated (Supplementary Fig. S8, p < 0.001). As a two-stage sensitivity analysis, a pooled random-effects meta-analysis was performed with the HR of each study, reaching similar results of no difference in survival (HR = 1.13; [95 % CI: 0.96–1.33]; p = 0.15; I^2^ = 52.5 %; Supplementary Fig. S9). Egger's test showed no evidence for publication bias (Supplementary Fig. S10; p = 0.29). The time dependent HR and dRMST showed a higher initial mortality in women, but it became a neutral hazard after approximately five years (Supplementary Fig. S11). A small number of patients needed reoperations over a 10-year period (1 % in both sexes), with no difference between groups (HR = 1.65; [95 % CI: 0.39–6.91]; p = 0.5; Fig. 3B). However, the hazard for recurrent mitral regurgitation was significantly higher in women over a 10-year period (HR 1.81; [95 % CI 1.08–2.37]; p = 0.02; Fig. 3C), occurring in 16 % of women and 10 % of men.

### 3.4 Subgroup analysis

**Fig. 4:**
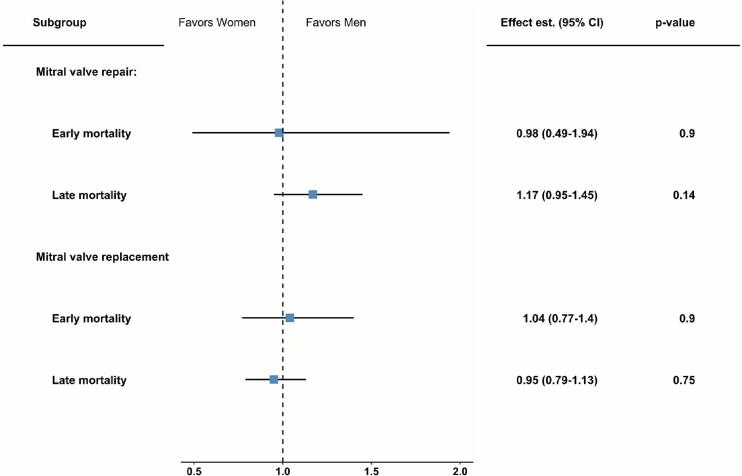
Subgroup analysis of MVr vs MVR. Similar short- and long-term mortality was observed among sexes independent of the surgical approach. **CI:** confidence interval; **Effect est.** = effect estimate - risk ratio for short-term mortality and hazard ratio for long-term; **MVr:** mitral valve repair; **MVR:** mitral valve replacement.

Outcomes in the subgroup analysis were consistent with the overall findings (Supplementary Figs. S12–13). Short-term mortality was similar among men and women undergoing MVr and MVR (p-value for interaction = 0.88). Long-term mortality was also similar in both sexes when treated with MVr or MVR (Fig. 4).

We conducted an additional subgroup analysis focusing on patients with degenerative mitral regurgitation and found a strong trend towards worse survival in women, but without statistical significance (HR 1.18; [95 % CI: 0.99–1.41]; p = 0.06; Supplementary Fig. S14).

### 3.5 Risk of bias

All studies were graded as being at moderate risk of bias (Supplementary Fig. S15). All studies were considered at moderate risk of bias in the first domain (confounding) due to their retrospective design. Three studies were rated as at moderate risk of selection bias due to issues in patient matching, and three studies showed concerns due to missing data in the databases used in their retrospective cohorts. No study was considered to be at a serious risk of bias.

### 3.6 Meta-regression

Among tested covariates, only sample size had a significant modulating effect for the early mortality outcome, reducing its risk (Table 3), but considering the very small coefficient, it might not be clinically significant. All variables adequately explained the heterogeneity for early mortality, but only baseline comorbidities (atrial fibrillation, hypertension, and diabetes) controlled the variation in late mortality.

**Table 3:** Meta regression of the mortality outcomes.

Variable	Short-term mortality			Long-term mortality		
	Coefficient (95 % CI)	p-value	Heterogeneity	Coefficient (95 % CI)	p-value	Heterogeneity
Age (years)	0.024 (−0.048–0.096)	0.51	I^2^ = 0 %; tau^2^ = 0	0.0023 (−0.07–0.08)	0.95	I^2^ = 38.51 %; tau^2^ = 0.08
Mitral valve repair (%)	0.0002 (−0.0062–0.0067)	0.94	I^2^ = 0 %; tau^2^ = 0	0.0023 (−0.0017–0.0063)	0.26	I^2^ = 11.29 %; tau^2^ = 0.038
Atrial fibrillation (%)	0.0005 (−0.0163–0.0172)	0.96	I^2^ = 0 %; tau^2^ = 0	−0.016 (−0.04–0.008)	0.18	I^2^ = 0 %; tau^2^ = 0
Hypertension (%)	0.0087 (−0.03–0.047)	0.66	I^2^ = 0 %; tau^2^ = 0	−0.0003 (−0.017–0.016)	0.97	I^2^ = 0 %; tau^2^ = 0
Diabetes (%)	0.0028 (−0.015–0.021)	0.76	I^2^ = 0 %; tau^2^ = 0	−0.009 (−0.027–0.0095)	0.33	I^2^ = 0 %; tau^2^ = 0
Sample Size	−0.00 (−0.00 to −0.00)	**0.005**	I^2^ = 0 %; tau^2^ = 0	0.00 (−0.00–0.0001)	0.48	I^2^ = 15 %; tau^2^ = 0.105
Follow-up time (years)	NA	NA	NA	−0.01 (−0.05–0.03)	0.20	I^2^ = 38.17 %; tau^2^ = 0.037

**CI:** confidence interval; **NA:** not applicable.

Statistically significant p-values are highlighted in bold.

### 3.7 Qualitative synthesis

No study reported on periprocedural myocardial infarction, deep sternal wound infection or surgical wound infection. Only two studies reported long-term heart failure hospitalization, stroke and myocardial infarction, finding similar outcomes in both groups [11,12]. Post-operative echocardiographic outcomes were reported by four studies [11,12,16,18], and are summarized in Supplementary Table S4. Implanted prosthesis size was similar in both groups in the study by El-Andari et al. [12], but larger in men in the study of Passos et al. [18]. Three studies reported mitral valve gradient, in two studies gradients were similar [11,12], but in one study women had higher gradients than men [18]. In the two studies reporting long-term left ventricle ejection fraction, both found a superior value in women [12,18]. Two studies reported trends towards more tricuspid regurgitation in women [16,18]. Changes in chamber size were reported by two studies, one noting similar body-indexed changes in left ventricle size and left atrium size and volume [11], while the other study observed more significant changes in left ventricle and atrium size in men, but changes in left atrium volume index were similar among groups [12].

## 4 Discussion

In this meta-analysis of 12 studies including 55,616 patients, we found that: (1) in the short term, mortality, stroke, kidney injury, postoperative atrial fibrillation, and permanent pacemaker implantation rates were similar between groups; and (2) in the long-term follow-up, mortality and reoperation rates were similar, while recurrent mitral regurgitation was more frequent in women.

El-Andari et al. conducted two of the studies in our analysis and also published the first and currently only meta-analysis on this topic [22]. They pooled data from 12 studies, matched and unmatched and found similar in-hospital/30-day mortality in men and women, but higher 1-year mortality in women. They acknowledge the lack of PSM studies and long-term data as limitations of their study, which are addressed by our present analysis. We similarly found no differences in early mortality, but we also found comparable long-term survival in both sexes. Additionally, we were able to perform isolated analysis for each surgical approach (repair or replacement) and also found comparable short- and long-term mortality. Female sex is considered a risk factor for in-hospital mortality in the Society of Thoracic Surgeons risk model for patients undergoing valve procedures [23]. Our results, as well as the previous meta-analysis, indicate no sex-based differences in early mortality following MVS. This discrepancy suggests that by itself female sex might not be a risk factor in patients with similar covariates, but rather a marker for worse baseline risk status.

Women have more anterior and bi-leaflet pathologies, tissue thickening, and mitral annulus calcification, which adds complexity to MVr, increases cross-clamping time and can result in higher mortality and an increased risk for recurrent mitral regurgitation [18]. Our findings indicate an important bridging of mortality, but when considering sex-based differences in life expectancy, it can be questioned if similar survival truly reflects outcome equality or if it underscores a worse result in women.

Some treatment differences observed in our review were: (1) a smaller proportion of women received MVr even after baseline-matching (23.8 % vs 35.7 %); (2) women are less likely to be considered for minimally invasive mitral valve procedures but undergo more transcatheter repair [3]; (3) even when presenting with more atrial fibrillation and tricuspid regurgitation, women do not receive more concomitant ablation and tricuspid replacement; and (4) when undergoing MVr, women receive less in situ chordal transfer due to a lack of enough healthy native chordae tendineae [15].

These differences in baseline pathology and in treatment pattern likely correlate with the observed higher hazard for recurrent mitral regurgitation in women and give a biological basis for this outcome. In particular, as women present with different leaflet pathology and more annular calcification, MVS could be considered to be more challenging, on average, in women, and require specific managing strategies. The importance of mitral regurgitation lies in its relation to MVr's ability to restore life expectancy in certain patients to values compatible with the overall population [24]. For this to happen, a durable repair is needed as the degree of regurgitation directly correlates with survival [24]. In other words, the repair quality and durability compares to “pharmaco-adherence” in medical therapy. Nevertheless, this outcome was pooled from only three studies and should be interpreted cautiously. A dedicated prospective study to assess the impact of patient sex on mitral regurgitation after MVS might be needed to provide better evidence.

The single-institution cohort by Malik indicates improved early referral of women for MVS after 2014, with overall lower NYHA class at the time of surgery, owing to increased awareness of sex-based disparities and efforts to improve outcomes. To further facilitate early intervention and alleviate referral bias, guideline-recommended cut-off values for treatment could be indexed to body size. Current Class I recommendations from the ESC/EACTS guidelines are based on left ventricular enlargement, resulting in fewer women meeting inclusion criteria [1]. As previously proposed, sex-specific left ventricular and atrial diameter cut-offs could help promote equal referral and intervention timing in both sexes [25].

### 4.1 Limitations

This study has important limitations to consider. As a meta-analysis of observational studies, there is a risk of confounding and selection bias, despite statistical adjustments and baseline matching, particularly given the retrospective nature of the included studies. While a meta-analysis of randomized trials is the gold standard, sex is not a randomizable variable, making such trials unfeasible. We did not have access to true patient-level data and instead relied on Kaplan-Meier-derived individual patient data, limiting our ability to perform more extensive subgroup analyses. We could not pool data on important secondary endpoints, such as deep sternal wound infection or periprocedural myocardial infarction, as well as echocardiographic outcomes (long-term mitral stenosis, valve gradients, or valve area) and valve-related events (patient prosthesis-mismatch, valve thrombosis, or endocarditis). Considering the discussed differences in valve disease and treatment pattern, as well as the reported echocardiographic endpoints in a limited number of studies, it could be possible for there to be an important difference in these outcomes. Lastly, there was significant variation in the populations of included studies regarding type of mitral valve disease, surgical approach, concomitant procedures, geographical distribution, and, consequently, healthcare settings. These variations are important as there might be sex-based differences in some subgroups depending on baseline disease or etiology. Chang reported better survival of women operated for rheumatic mitral disease [10], while in our study we found a strong trend towards worse survival in women receiving MVS for degenerative mitral disease.

## 5 Conclusion

In this systematic review and meta-analysis, we assessed the impact of sex on the outcomes of MVS. Addressing the data from 55,616 patients, we found no significant sex-based differences in short-term mortality or complications, as well as in long-term mortality or reoperation. A higher hazard of recurrent mitral regurgitation was observed in women in a subset of three studies, indicating a potentially less durable repair. This finding should be interpreted with caution given the limited evidence and highlights the need for further prospective studies to confirm it.

## CRediT authorship contribution statement

**Leo Consoli:** Writing – review & editing, Writing – original draft, Project administration, Methodology, Conceptualization. **Mir W. Majeed:** Writing – review & editing, Writing – original draft, Data curation, Conceptualization. **Eren Cetinel:** Visualization, Validation, Investigation. **Pawel Lajczak:** Validation, Software, Formal analysis. **Ilias G. Koziakas:** Visualization, Validation, Investigation. **Hristo Kirov:** Writing – review & editing, Validation, Formal analysis. **Torsten Doenst:** Writing – review & editing, Supervision, Data curation, Conceptualization. **Tulio Caldonazo:** Writing – review & editing, Supervision, Funding acquisition, Formal analysis.

## Funding

T.C. was funded by the 10.13039/501100001659Deutsche Forschungsgemeinschaft (DFG, German Research Foundation) Clinician Scientist Program OrganAge funding number 413668513, by the 10.13039/501100005971Deutsche Herzstiftung (DHS, German Heart Foundation) funding number S/03/23 and by the Interdisciplinary Center of Clinical Research of the Medical Faculty Jena.

## Declaration of competing interest

The authors declare that they have no known competing financial interests or personal relationships that could have appeared to influence the work reported in this paper.

## Data Availability

The data generated in this article is available in the article and in its online supplementary material. The raw data used in the analysis is publicly available in the manuscripts of the included studies.

## Appendix A Supplementary data

Supplementary material Image 1
