# Supplementary material for: Sex-based differences in outcomes of mitral valve surgery: A meta-analysis of propensity score-matched studies with reconstructed time-to-event data
Source: Am Heart J Plus. 2025 Nov 21;61:100682. doi: 10.1016/j.ahjo.2025.100682 (PMC12682065; doi:10.1016/j.ahjo.2025.100682)
Supplement: Supplementary file 1 — Supplementary material [file mmc1.docx]

**Supplementary material**

**Table of contents**

**Appendix 1: PRISMA checklist .............................................................................................2 Appendix 2: Search strategy.……………………..…............................................................4 Appendix 3: Outcome definitions……..…………................................................................ 5 Appendix 4: Statistical methods…………………………………………………………….6 Appendix 5: Additional tables…………………………………………………………….…7 Appendix 6: Additional figures……….………………………...…………………………...9**

| **Section and**  **Topic** | **Item #** | **Checklist item** | **Location where item is reported** |
| --- | --- | --- | --- |
| **TITLE** | | |  |
| Title | 1 | Identify the report as a systematic review. | Page 1 |
| **ABSTRACT** | | |  |
| Abstract | 2 | See the PRISMA 2020 for Abstracts checklist. | Page 2 |
| **INTRODUCTION** | | |  |
| Rationale | 3 | Describe the rationale for the review in the context of existing knowledge. | Page 5 |
| Objectives | 4 | Provide an explicit statement of the objective(s) or question(s) the review addresses. | Page 5 |
| **METHODS** | | |  |
| Eligibility criteria | 5 | Specify the inclusion and exclusion criteria for the review and how studies were grouped for the syntheses. | Page 6 |
| Information sources | 6 | Specify all databases, registers, websites, organizations, reference lists and other sources searched or consulted to identify studies. Specify the date when each source was last searched or consulted. | Page 6 |
| Search strategy | 7 | Present the full search strategies for all databases, registers and websites, including any filters and limits used. | Supplement |
| Selection process | 8 | Specify the methods used to decide whether a study met the inclusion criteria of the review, including how many reviewers screened each record and each report retrieved, whether they worked independently, and if applicable, details of automation tools used in the process. | Page 6 |
| Data collection process | 9 | Specify the methods used to collect data from reports, including how many reviewers collected data from each report, whether they worked independently, any processes for obtaining or confirming data from study investigators, and if applicable, details of automation tools used in the process. | Page 7 |
| Data items | 10a | List and define all outcomes for which data were sought. Specify whether all results that were compatible with each outcome domain in each study were sought (e.g. for all measures, time points, analyses), and if not, the methods used to decide which results to collect. | Page 7 |
|  | 10b | List and define all other variables for which data were sought (e.g. participant and intervention characteristics, funding sources). Describe any assumptions made about any missing or unclear information. | Page 7 |
| Study risk of bias assessment | 11 | Specify the methods used to assess risk of bias in the included studies, including details of the tool(s) used, how many reviewers assessed each study and whether they worked independently, and if applicable, details of automation tools used in the process. | Page 7 |
| Effect measures | 12 | Specify for each outcome the effect measure(s) (e.g. risk ratio, mean difference) used in the synthesis or presentation of results. | Page 7 |
| Synthesis methods | 13a | Describe the processes used to decide which studies were eligible for each synthesis (e.g. tabulating the study intervention characteristics and comparing against the planned groups for each synthesis (item #5)). | Page 7-8 |
|  | 13b | Describe any methods required to prepare the data for presentation or synthesis, such as handling of missing summary statistics, or data conversions. | Page 7-8 |
|  | 13c | Describe any methods used to tabulate or visually display results of individual studies and syntheses. | Page 7-8 |
|  | 13d | Describe any methods used to synthesize results and provide a rationale for the choice(s). If meta-analysis was performed, describe the model(s), method(s) to identify the presence and extent of statistical heterogeneity, and software package(s) used. | Page 7-8 |
|  | 13e | Describe any methods used to explore possible causes of heterogeneity among study results (e.g. subgroup analysis, meta-regression). | Page 7-8 |
|  | 13f | Describe any sensitivity analyses conducted to assess robustness of the synthesized results. | Page 7-8 |
| Reporting bias assessment | 14 | Describe any methods used to assess risk of bias due to missing results in a synthesis (arising from reporting biases). | Page 7-8 |
| Certainty assessment | 15 | Describe any methods used to assess certainty (or confidence) in the body of evidence for an outcome. | N/A |
| **RESULTS** | | |  |
| Study selection | 16a | Describe the results of the search and selection process, from the number of records identified in the search to the number of studies included in the review, ideally using a flow diagram. | Page 8 |
|  | 16b | Cite studies that might appear to meet the inclusion criteria, but which were excluded, and explain why they were excluded. | Supplement |
| Study characteristics | 17 | Cite each included study and present its characteristics. | Page9 / Table 1 |
| Risk of bias in studies | 18 | Present assessments of risk of bias for each included study. | Page 11 |
| Results of individual studies | 19 | For all outcomes, present, for each study: (a) summary statistics for each group (where appropriate) and (b) an effect estimate and its precision (e.g. confidence/credible interval), ideally using structured tables or plots. | Forest plots (Figure 2) |
| Results of syntheses | 20a | For each synthesis, briefly summarise the characteristics and risk of bias among contributing studies. | Pages 9-10 |
|  | 20b | Present results of all statistical syntheses conducted. If meta-analysis was done, present for each the summary estimate and its precision (e.g. confidence/credible interval) and measures of statistical heterogeneity. If comparing groups, describe the direction of the effect. | Pages 9-10 |
|  | 20c | Present results of all investigations of possible causes of heterogeneity among study results. | Pages 9-10 |
|  | 20d | Present results of all sensitivity analyses conducted to assess the robustness of the synthesized results. | Pages 9-10 |
| Reporting biases | 21 | Present assessments of risk of bias due to missing results (arising from reporting biases) for each synthesis assessed. | Pages 10 |
| Certainty of evidence | 22 | Present assessments of certainty (or confidence) in the body of evidence for each outcome assessed. | N/A |
| **DISCUSSION** | | |  |
| Discussion | 23a | Provide a general interpretation of the results in the context of other evidence. | Page 11-12 |
|  | 23b | Discuss any limitations of the evidence included in the review. | Page 13 |
|  | 23c | Discuss any limitations of the review processes used. | Page 13 |
|  | 23d | Discuss implications of the results for practice, policy, and future research. | Page 11-13 |
| **OTHER INFORMATION** | | |  |
| Registration and protocol | 24a | Provide registration information for the review, including register name and registration number, or state that the review was not registered. | Page 5 |
|  | 24b | Indicate where the review protocol can be accessed, or state that a protocol was not prepared. | Page 5 |
|  | 24c | Describe and explain any amendments to information provided at registration or in the protocol. | N/A |
| Support | 25 | Describe sources of financial or non-financial support for the review, and the role of the funders or sponsors in the review. | Page 14 |
| Competing interests | 26 | Declare any competing interests of review authors. | Page 14 |
| Availability of data, code and other materials | 27 | Report which of the following are publicly available and where they can be found: template data collection forms; data extracted from included studies; data used for all analyses; analytic code; any other materials used in the review. | Page 14 |

*From:*  Page MJ, McKenzie JE, Bossuyt PM, Boutron I, Hoffmann TC, Mulrow CD, et al. The PRISMA 2020 statement: an updated guideline for reporting systematic reviews. BMJ 2021;372:n71. doi: 10.1136/bmj.n71. This work is licensed under CC BY 4.0. To view a copy of this license, visit <https://creativecommons.org/licenses/by/4.0/>

**Appendix 2: Search Strategy**

Available below is the search strategy we created for PubMed and modified for the other databases as was necessary using relevant terms and syntax. All references from selected studies were also manually retrieved for “backwards snowballing”.

**PubMed search strategy:** (mitral valve surgery OR “mitral valve replacement” OR “mitral valve repair”) AND ("sex differences" OR "gender disparities" OR "gender differences" OR "sex-specific outcomes" OR "sex-related differences" OR “sex-based differences”)

**Embase search strategy:** (mitral valve surgery OR “mitral valve replacement” OR “mitral valve repair”) AND ("sex differences" OR "gender disparities" OR "gender differences" OR "sex-specific outcomes" OR "sex-related differences" OR “sex-based differences”)

**Cochrane search strategy:** (mitral valve surgery OR “mitral valve replacement” OR “mitral valve repair”) AND ("sex differences" OR "gender disparities" OR "gender differences" OR "sex-specific outcomes" OR "sex-related differences" OR “sex-based differences”)

**Applied filters:** Search date was restricted from 2013 up to 2025.

**Appendix 3: Outcome definitions**

**Supplementary Table S1**: Outcome definitions.

| **Outcome** | **Definition** |
| --- | --- |
| **Early mortality** | Any in-hospital death or up to 30 days after surgery |
| **Kidney injury** | New-onset renal failure (KDIGO criteria) during hospital stay or up to 30 days after surgery. |
| **Stroke** | Any stroke diagnosed by an imaging method during hospital stay or up to 30 days after surgery. |
| **Postoperative atrial fibrillation** | Atrial fibrillation lasting for at least 30s diagnosed during hospital stay or up to 30 days after surgery. |
| **Permanent Pacemaker** | Implantation of a new pacemaker during hospital stay or up to 30 days after surgery. |
| **Late mortality** | All-cause mortality at least 1 year after surgery. |
| **Late reoperation** | Mitral valve reoperation (surgical or transcatheter) at least 1 year after the primary procedure. |
| **Late recurrent mitral regurgitation** | Onset of mitral regurgitation +3 or symptoms of mitral regurgitation at least 1 year after surgery. |

**Appendix 4 - Statistical methods:**

**Pairwise meta-analysis of short-term outcomes:**

We collected the number of events and total number of patients in the exposure (female) and control (male) groups. This data was stored into a prespecified Excel spreadsheet and imported into R studio. We pooled studies in a random-effects inverse-variance meta-analysis with the “meta” package, obtaining Risk Ratios and 95% confidence intervals. Heterogeneity estimators were determined using Der Simonian-Laird method. To understand how heterogeneity impacted the distribution of effect size estimates, a prediction interval was also calculate for all endpoints. A subgroup analysis was done comparing mitral valve repair versus replacement.

**Reconstructing individual patient data (IPD):**

Kaplan-Meier curves were digitalized and imported into the ShinyApp web software. Survival probabilities at multiple time points were extracted and combined with the numbers at risk to reconstruc individual-patient-data using the IPDfromKM method. This data from all studies was combined into a csv file and imported into R studio.

**Analysis of the reconstructed IPD:**

We used the following packages: “survival”, “ggsurvfit”, “survRM2” and “rms”. Initially we tested the proportional hazards assumption with Grambsch-Thernau test and Schoenfeld residuals plot. If it was confirmed, a frailty Cox regression model was applied to obtain hazards ratios and confidence intervals. “Sex” was used as a fixed term (men and women) and “study” as the frailty term (random-effects), modeled as random intercepts. If the assumption was violated, the difference in restricted mean survival time was extracted from each study and pooled in a random-effects inverse variance meta-analysis with the “metafor” package, as this method has been show to be more accurate than simply extracting it from the overall reconstructed Kaplan-Meier curve. Lastly, splines were used with the “survRM2” package to model the time dependent hazards ratio. A subgroup analysis was conducted for long-term mortality comparing repair and replacement.

**Meta-regression:**

A mixed-effects meta-regression was done for important covariates concerning the mortality outcomes. Covariates were tested separately in pairs, adjusting for study sample size to account for small-study effects and reduce the risk of overfitting the model considering the relatively low number of observations. Each model included the covariate of interest and sample size, with a total of 8 variables.

**Publication bias:**

If at least 10 studies were present for a variable, funnel plot asymmetry was tested with Egger’s regression test.

**Appendix 5: Additional Tables**

**Supplementary Table S2:** Excluded fully-read studies and justifications

| **Author; Year** | **DOI or link** | **Reason for exclusion** |
| --- | --- | --- |
| Lancellotti 2024 | 10.1093/eurheartj/ehae524 | Unmatched study |
| Mantovani 2014 | 10.1159/000362180 | Unmatched study |
| Abadie 2024 | 10.1016/j.jacc.2023.10.033 | Unmatched study |
| Segar 2022 | 10.1002/ccd.30196 | Transcatheter mitral replacement |
| Giustino 2019 | 10.1016/S0735-1097(19)32557-4 | Unmatched study |
| Nappi 2022 | 10.1161/circ.146.suppl_1.9397 | Unmatched study |
| Al-Zubaidi 2023 | 10.59958/hsf.6741 | Unmatched study |
| Kim 2024 | 10.1093/eurheartj/ehae666.1792 | Unmatched study |
| Namazi 2021 | 10.1002/ehf2.13503 | Unmatched study |
| Grayburn 2016 | 10.1016/j.jcmg.2016.02.013 | Editorial |
| Johnston 2019 | 10.1161/JAHA.119.013260 | Unmatched study |
| Bradley 2022 | 10.1213/ANE.0000000000006076 | Unmatched study |
| Kataria 2019 | 10.1161/circ.140.suppl_1.14028 | Unmatched study |
| Tersalvi 2019 | 10.3390/medicina59061017 | Review article |
| Chang 2022 | 10.1136/bmjopen-2021-058538 | Population overlaps with Chang 2024 |
| Qi 2022 | 10.1136/bmjopen-2022-060882 | Unmatched study |
| Abadie 2025 | 10.1016/j.acvd.2024.10.156 | Unmatched study |
| Altes 2024 | 10.1016/j.jacadv.2024.101023 | Unmatched study |
| El-Andari 2021 | 10.1111/jocs.15431 | Editorial |
| Desmarais 2024 | 10.1007/s13670-024-00419-6 | Review article |
| Vakamudi 2018 | 10.1161/CIRCULATIONAHA.118.035789 | Unmatched study |
| Tran 2015 | 10.1097/HCO.0000000000000150 | Review article |

**Supplementary Table S3:** Baseline and procedural characteristics of individual studies

| **Study; Year** | **Age**^†^**, (F/M)** | **MVr, %**  **(F/M)** | **LVEF**^†^**,**  **(F/M)** | **Diabetes, % (F/M)** | **AF, %**  **(F/M)** | **HTN, % (F/M)** | **BMI**^†^**,**  **(F/M)** | **CAD, % (F/M)** | **DM, % (F/M)** | **CPB time**^†^**, (F/M)** | **ACC time**^†^**, (F/M)** |
| --- | --- | --- | --- | --- | --- | --- | --- | --- | --- | --- | --- |
| Chang; 2024 | 60.8***** | 20.5/30.6 | NA | 23.7***** | 43.8/43.4 | 51/57 | NA | 39.8/39.6 | 23.7/23.7 | NA | NA |
| El Andari; 2020 | 64.3/64.4 | None | 53.4/52 | 22.5***** | 14.8/16.1 | 65.9/67.8 | 28.3/28.4 | NA | 22.5/22.5 | 171.3/179.4 | 132.6/139.9 |
| El Andari; 2021 | 62.5/61.9 | All | 57.8/57.7 | 7.4/8 | 6.4/4.8 | 54.8***** | 26.6/26.8 | NA | 7.4/8 | 129.9/130.7 | 103.1/104 |
| Munoz-Rivas; 2020 | 64.2/64.1 | None | NA | 13.5/14.5 | 52.7/48.7 | NA | NA | NA | 12/14 | NA | NA |
| Kandula; 2021 | 60.9/61 | 98***** | 61.7/62.3 | 3/4 | 23/24 | 43***** | 25.5/25.6 | 16/13 | 3/4 | 85.17/89.33 | 72/74.17 |
| Kislitsina; 2019 | 59.6/59.9 | 98/96 | 61.6/61.2 | 2/2 | 17/18 | 41/42 | 25.5/25.6 | NA | 2/2 | 88.1/90.6 | 73.6/75.2 |
| Liu; 2023 | 56.5/56.7 | All | 63.3/63.3 | 7.6/8.8 | 34.4/34.1 | 41.4/38.4 | 24.32/24.38 | 7.6/9.1 | 24.32/24.38 | 103.21/109.45 | 73.73/77.44 |
| Malik; 2024 | 68.8/63.7 | All | 65/60 | 6.1/8.2 | 28.6/24.8 | NA | 25.46/26.59 | 8.2/14 | 6.1/8.2 | 134/145 | 97.92/104 |
| Passos; 2023 | 65.8/65.1 | 81.2/90.1 | 64.2/64.1 | NA | 16.8/17.8 | 31.7/29.7 | NA | 14.9/13.9 | NA | 162/167 | 104/108 |
| Saijo; 2024 | 72***** | 1***** | NA | 49/51 | 35/42 | 85/86 | 30.7/30.0 | NA | 49/51 | NA | NA |
| Long; 2025 | 63.7***** | 88/94 | NA | NA | NA | NA | 25.6/25.5 | 11/13 | 7.9/5.3 | 109.3/126.7 | 79.7/93.3 |
| Bernard; 2023^&^ | NA | 35.8/54.5 | NA | NA | NA | NA | NA | NA | NA | NA | NA |

**&:** Abstract only; **†:** mean; ***:** mean or percentage of both groups combined; **F:** female; **M:** male; **NA:** not available; **MVr:** Mitral Valve repair; **LVEF:** Left Ventricular Ejection Fraction; **AF**: Atrial Fibrillation; **HTN:** Hypertension.

**Supplementary Table S4:** Post-operative echocardiographic findings of individual studies

| **Study** | **Reported findings** |
| --- | --- |
| El-Andari, 2020 | Similar residual moderate-severe MR at 1-year / Similar body-indexed changes in LV size and in LA size and volume index / Similar mitral valve gradient and areas. |
| El-Andari, 2021 | Indexed changes in LV and LA size were greater in men, while absolute changes in LA volume index were similar / Similar residual moderate-severe MR at 4-months / Superior post-operative LVEF in women at 4-months / Similar mitral valve gradient and areas / Similar implanted prosthesis size. |
| Liu, 2023 | More females than males developed tricuspid regurgitation after surgery (p-value not provided). |
| Passos, 2023 | Larger ring size in valve repair and implanted prosthesis size in men compared to women / Similar post-operative LVEF, MR > 2, mitral valve gradients, and tricuspid regurgitation > 2 / Superior long-term LVEF in women / Trend towards more long-term tricuspid regurgitation > 2 in women / Similar long-term MR > 2 / Higher long-term mitral valve gradients in women. |

**MR:** mitral regurgitation; **LV:** left ventricle; **LA:** left atrium; **LVEF**: left ventricle ejection fraction;

**Appendix 6 - Additional figures:**

**
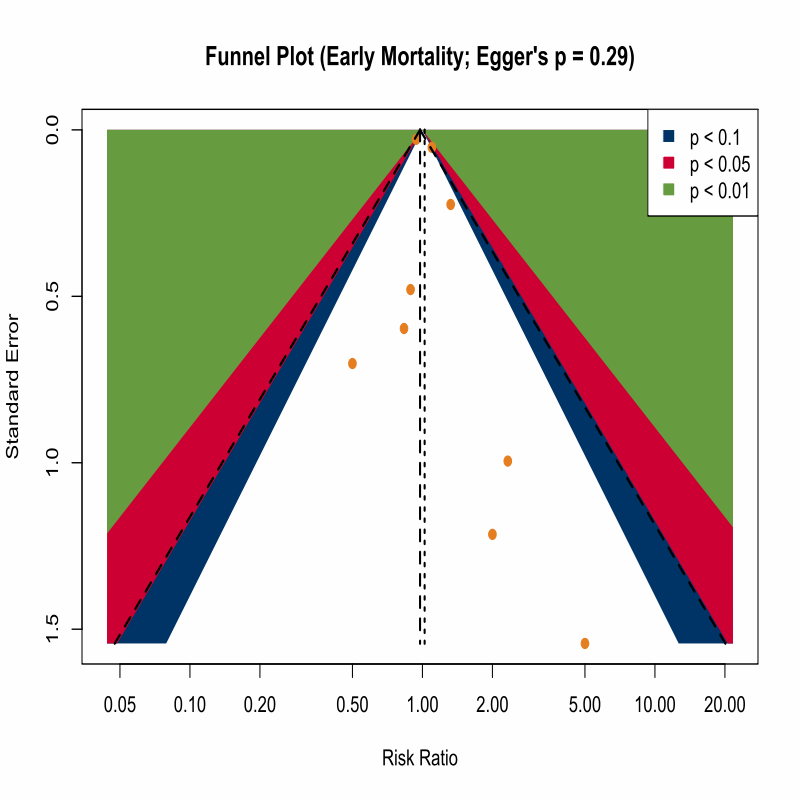
**

**Supplementary Figure S1**: Contour-enhanced funnel plot for early mortality. Egger’s regression test for funnel plot asymmetry was negative (p = 0.29).

**
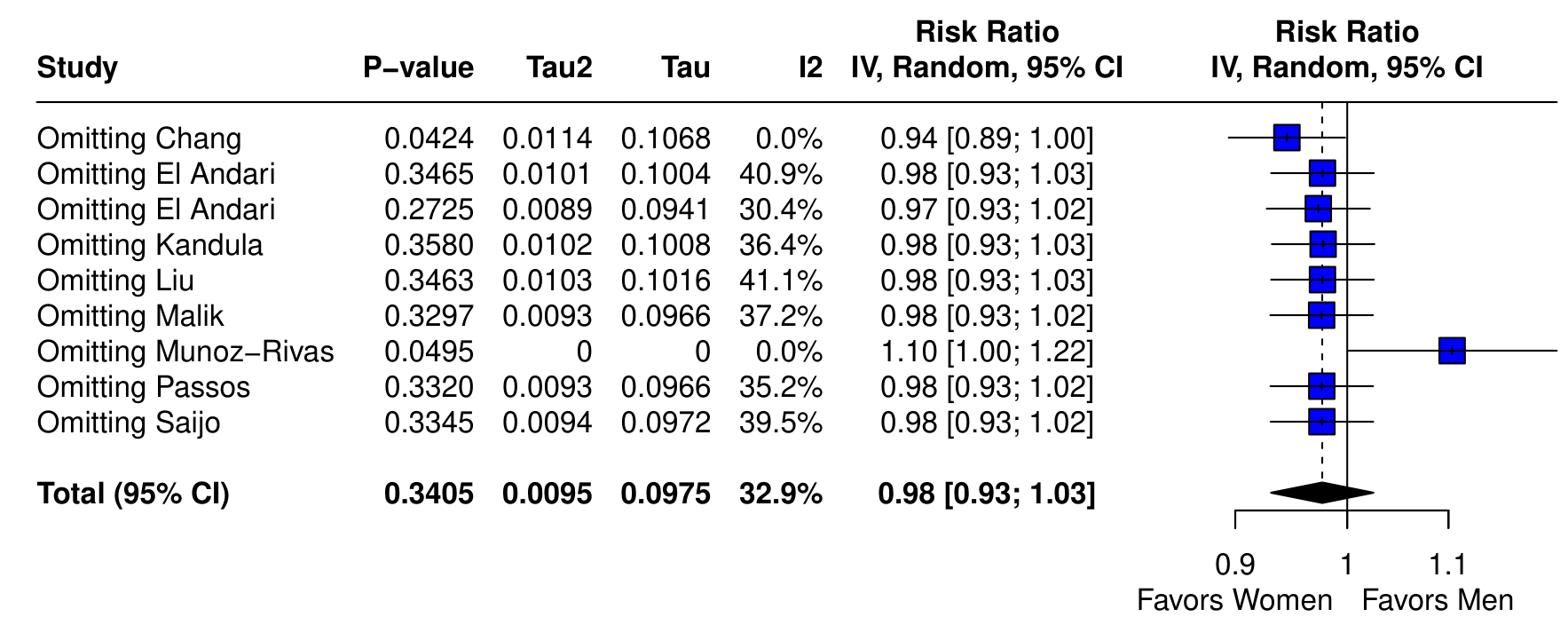
**

**Supplementary Figure S2:** Leave-one-out analysis for the outcome of short-term mortality showing that the effect estimate could change direction if the study by Chang or Munoz-Rivas were to be removed.

**
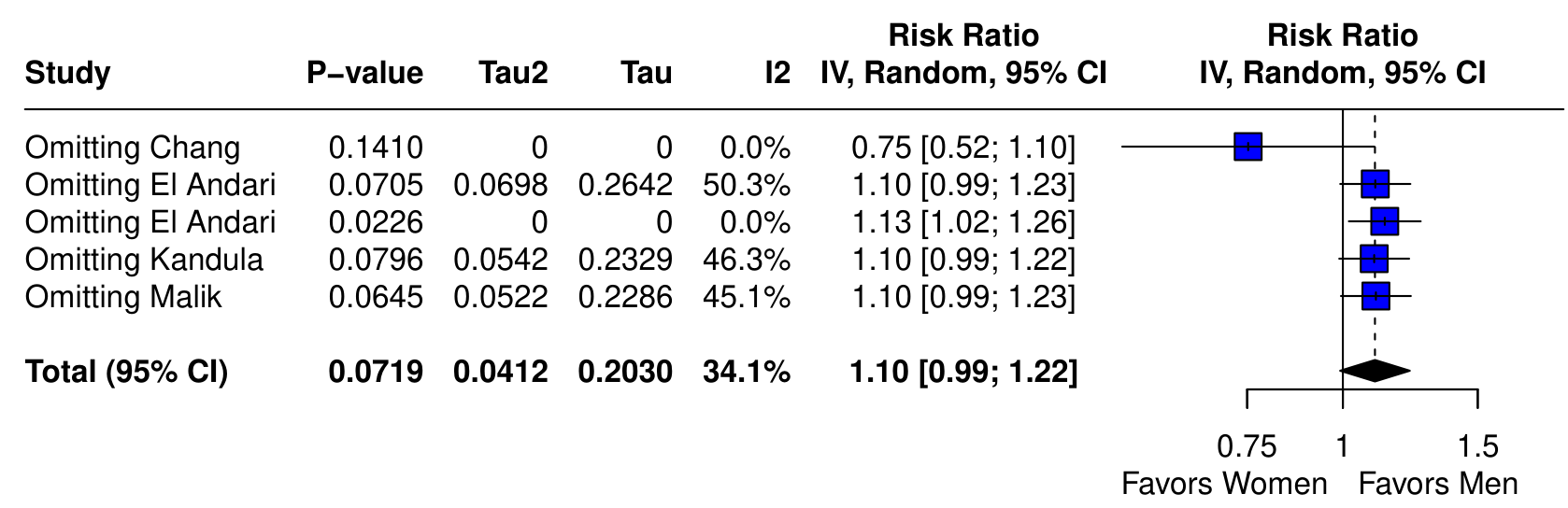
**

**Supplementary Figure S3:** Leave-one-out analysis for the outcome of kidney injury. Removal of most studies would move the effect estimate towards favoring men, indicating poor stability.


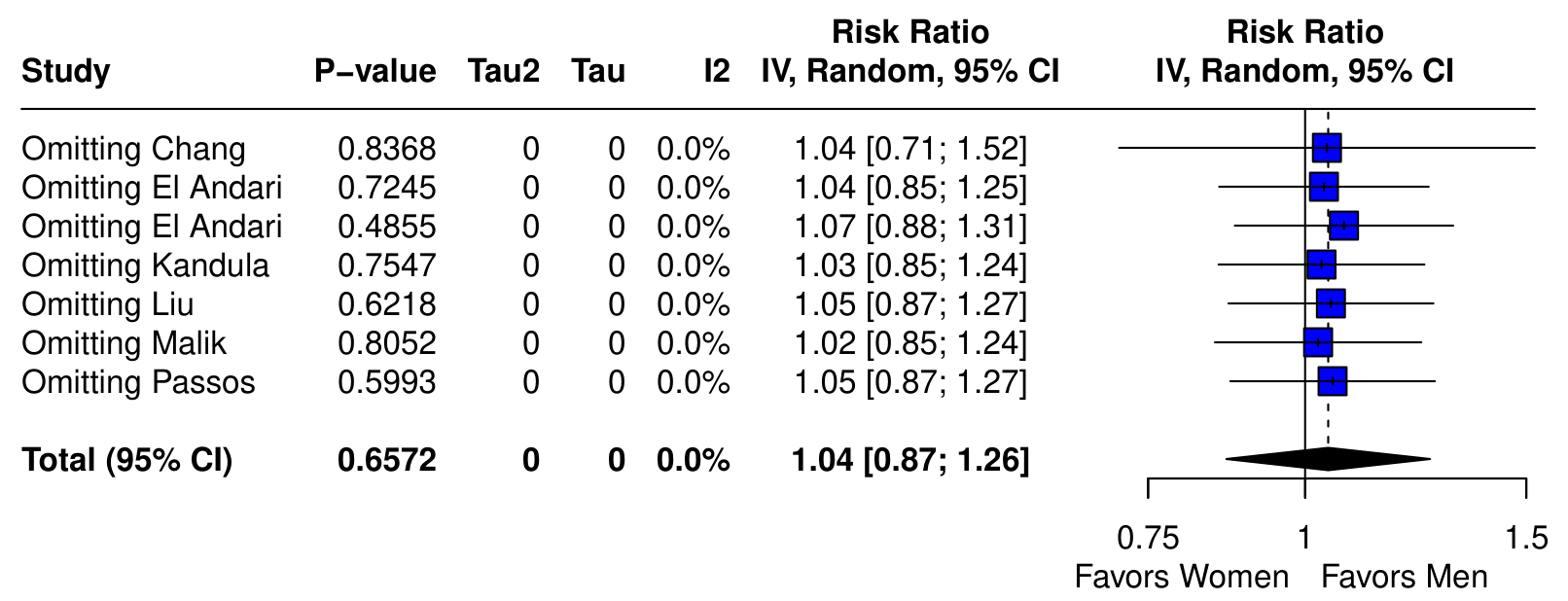


**Supplementary Figure S4:** Leave-one-out analysis for the outcome of stroke. The removal of no single study markedly changed the effect estimate.


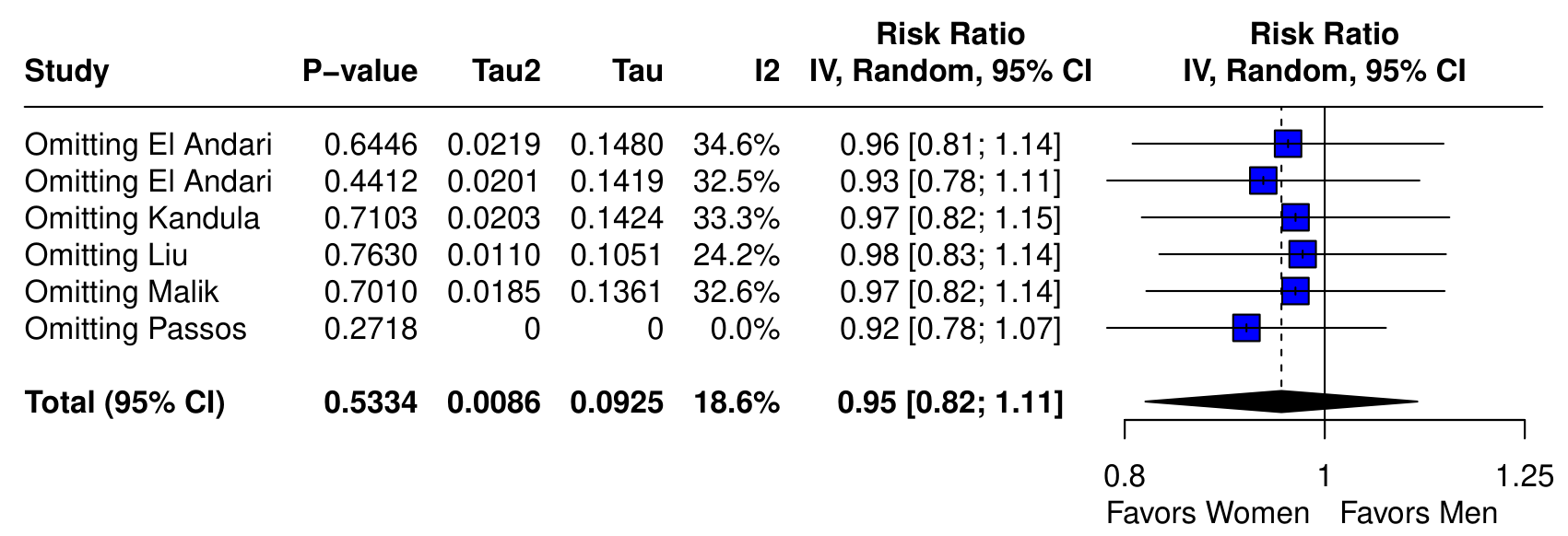


**Supplementary Figure S5:** Leave-one-out analysis for the outcome of postoperative atrial fibrillation. The removal of no single study markedly changed the effect estimate.


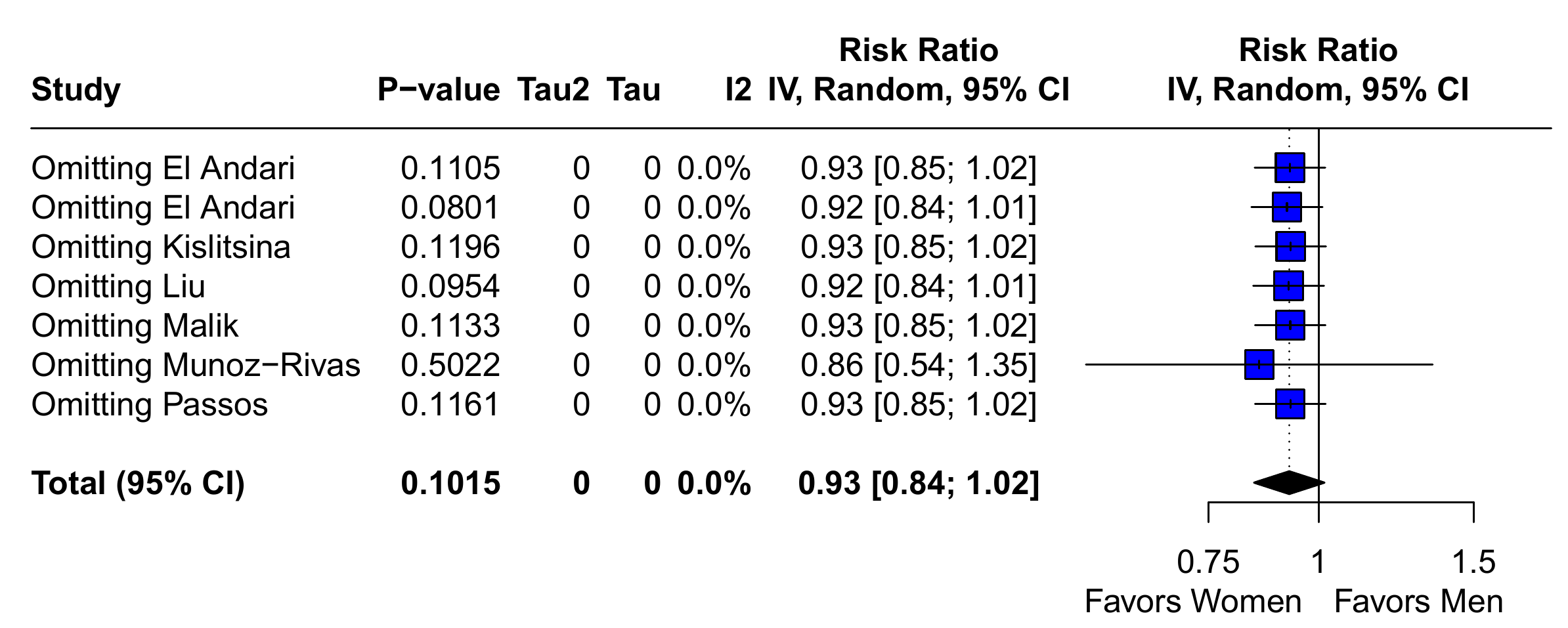


**Supplementary Figure S6:** Leave-one-out analysis for the outcome of pacemaker implantation.


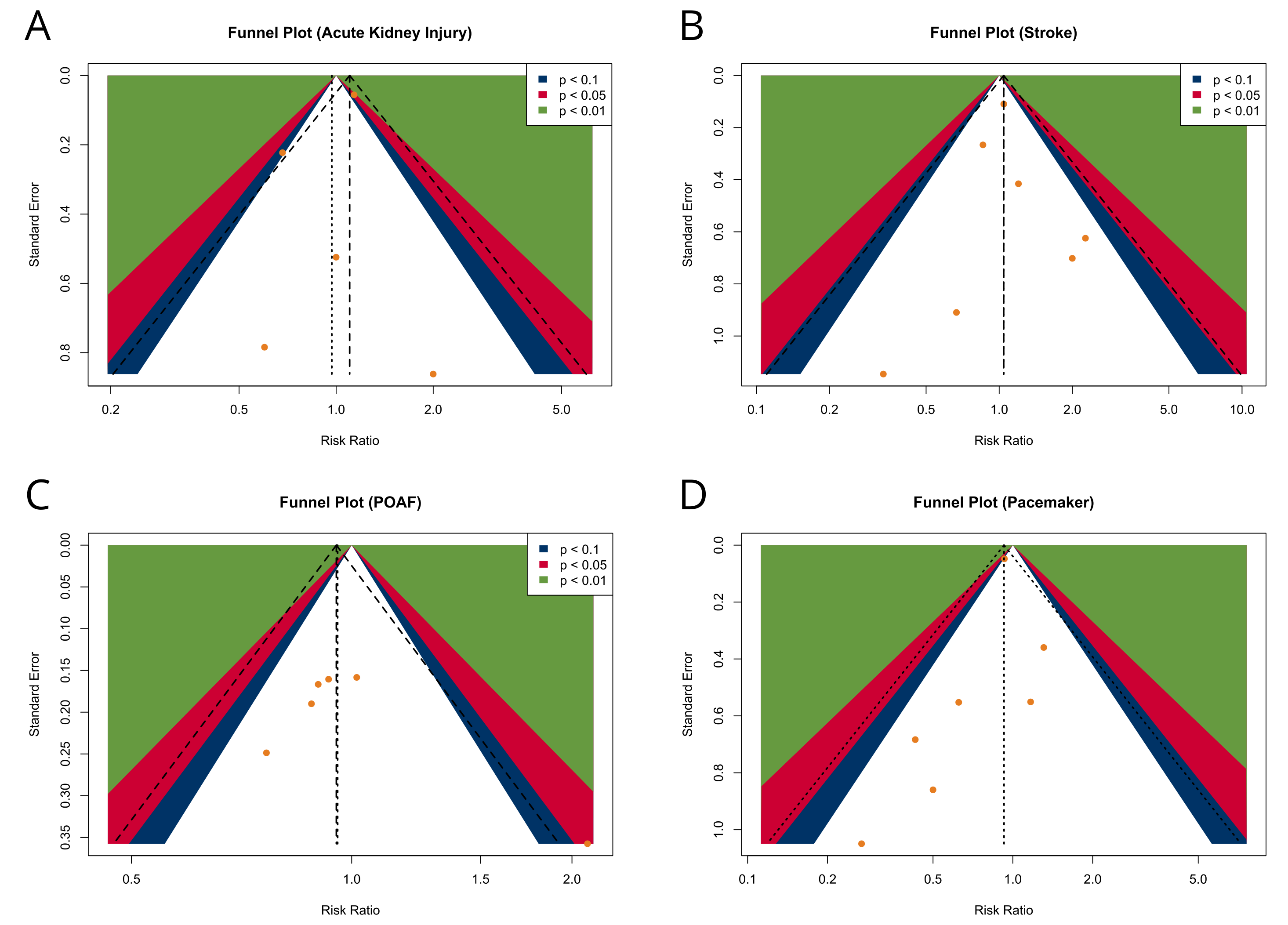


**Supplementary Figure S7:** Funnel plots of the short-term pairwise outcomes. **(A)**: kidney injury; **(B):** stroke; **(C):** atrial fibrillation; and **(D):** pacemaker.


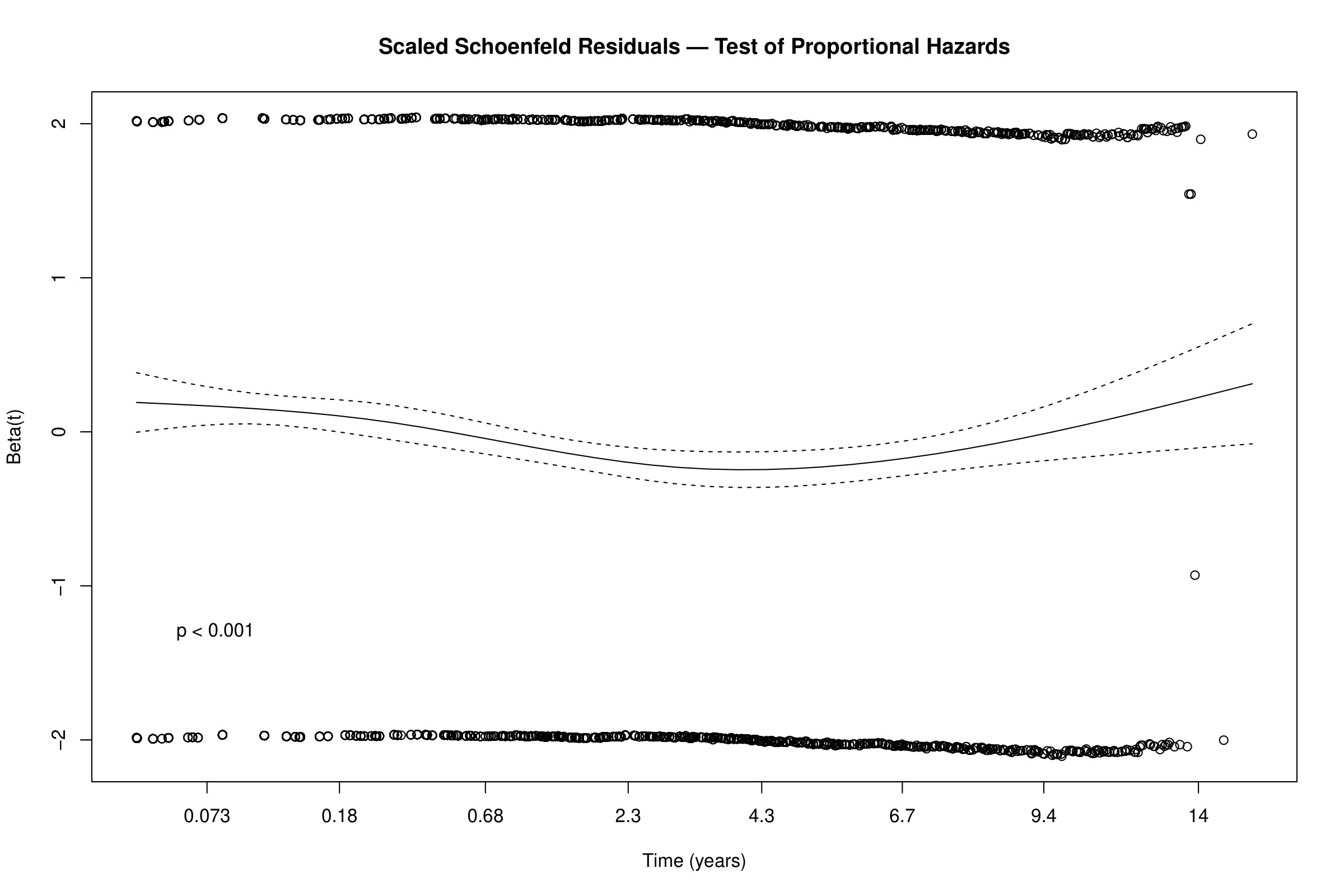


**Supplementary Figure S8:** Scaled Schoenfeld residuals plot. The test of proportional hazards indicate non-proportional hazards for the outcome of all-cause mortality (p < 0.001).

**
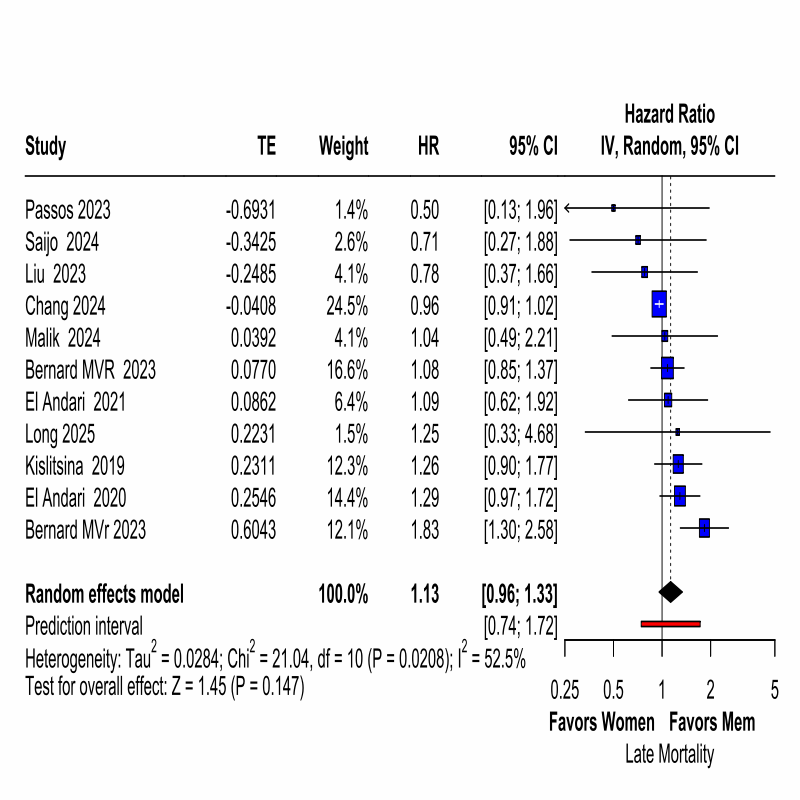
**

**Supplementary Figure S9:** Pooled random-effects inverse variance meta-analysis of the study reported hazard ratios for long-term mortality, showing comparable results to what was found from the IPDfromKM method, indicating good robustness for this outcome.


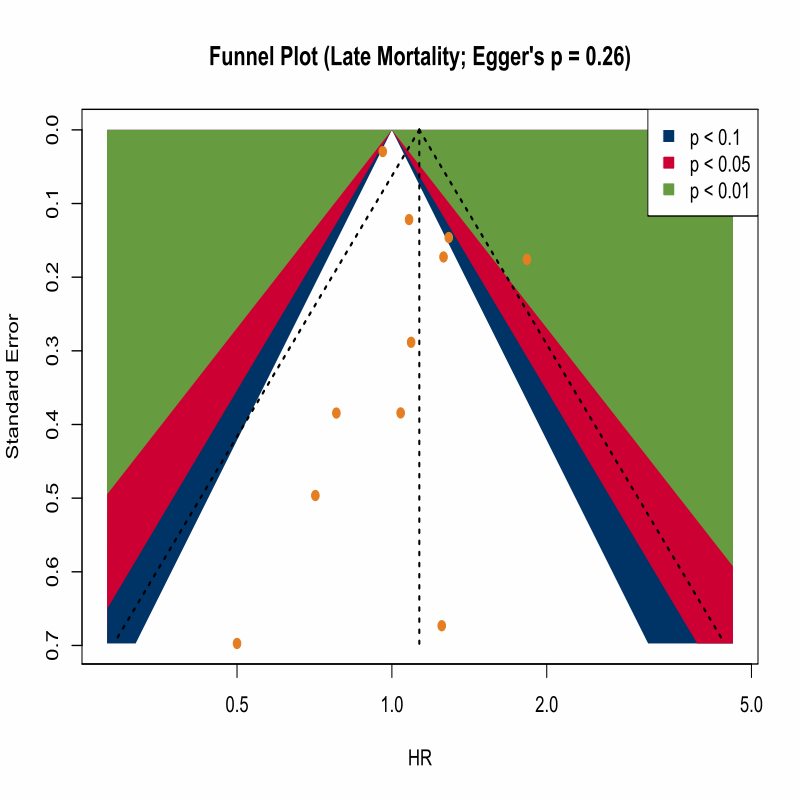


**Supplementary Figure S10**: Contour-enhanced funnel plot for late mortality. Despite asymmetry on visual inspection, Egger’s regression test for funnel plot asymmetry was negative (p = 0.26).


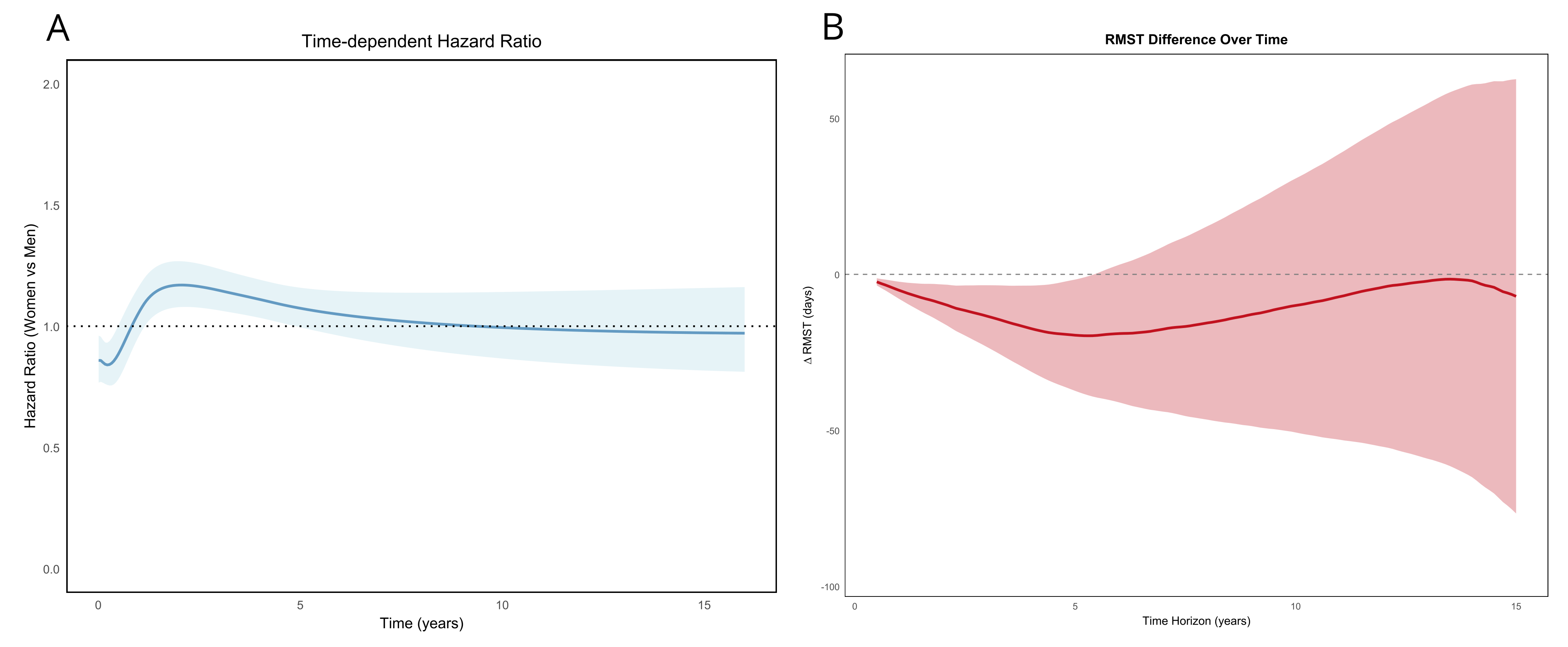


**Supplementary Figure S11**: **(A):** Time-dependent hazard ratio of women vs men over time in years of long-term mortality; **(B):** difference in restricted mean survival time of long-term mortality. The shaded blue and red ares represent the 95% confidence interval.


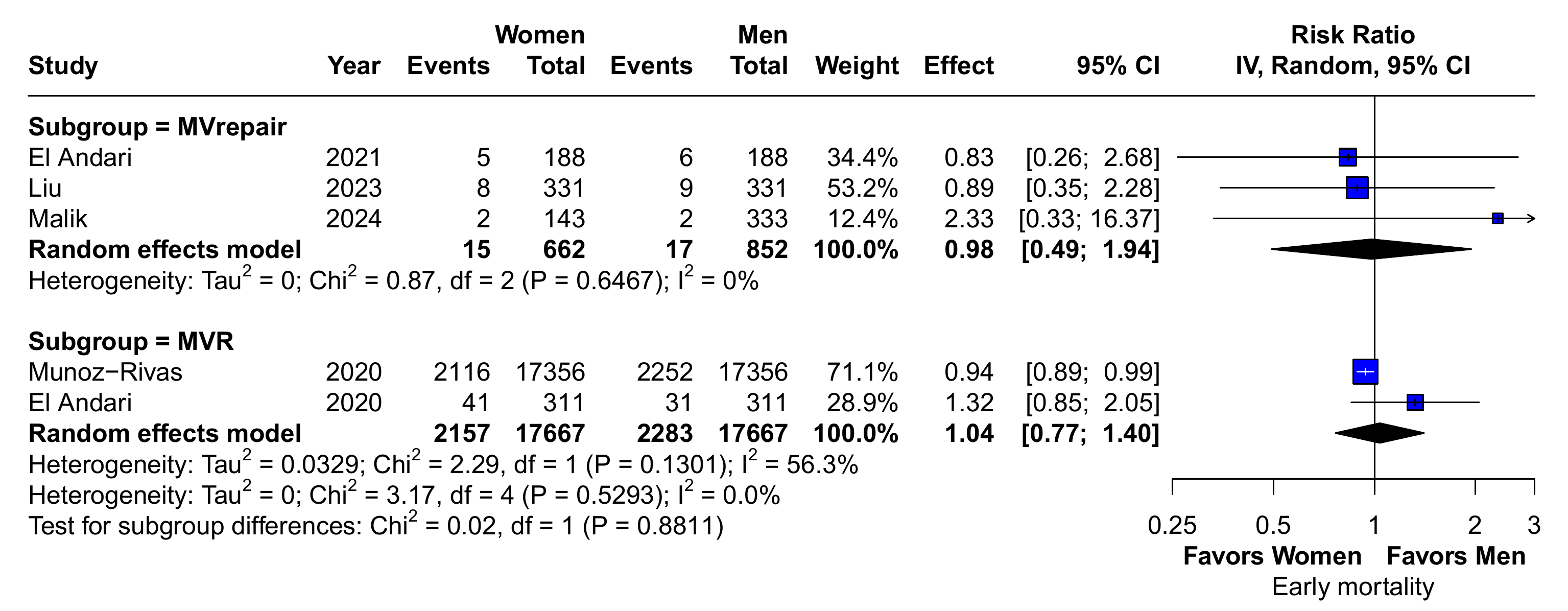


**Supplementary Figure S12**: Subgroup analysis of early mortality isolated by type of surgery, mitral valve repair and replacement, showing comparable mortality among sexes. However, the limited number of studies diminishes the power of this analysis.


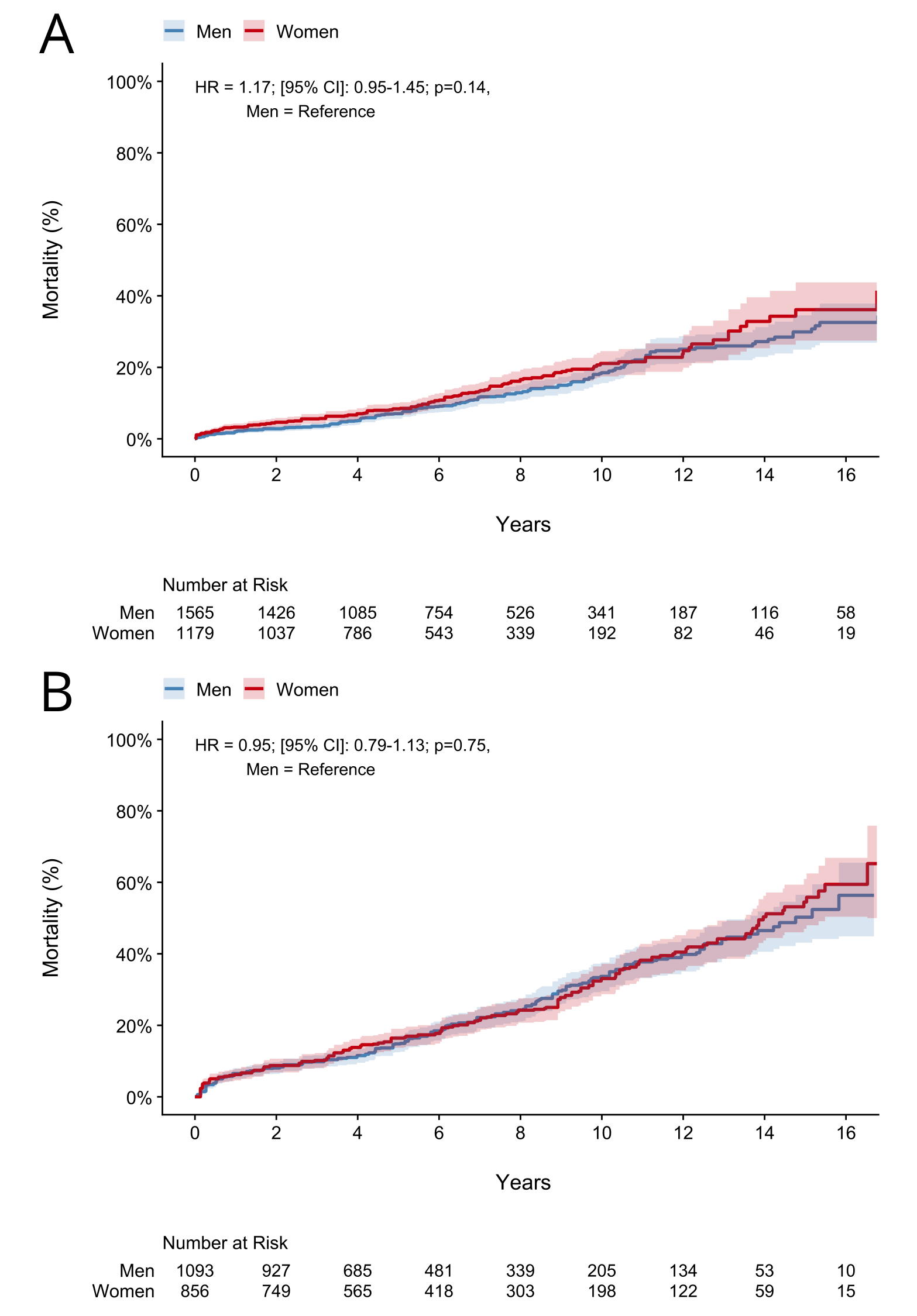


**Supplementary Figure S13**: **(A):** Subgroup analysis of long-term mortality in patients undergoing mitral valve repair showing similar survival in both groups. **(B):** Subgroup analysis of long-term mortality in patients undergoing mitral valve replacement showing similar survival in both groups. It can also be appreciated that mortality was higher in the replacement subgroup, which can be expected from the literature comparing these two approaches.


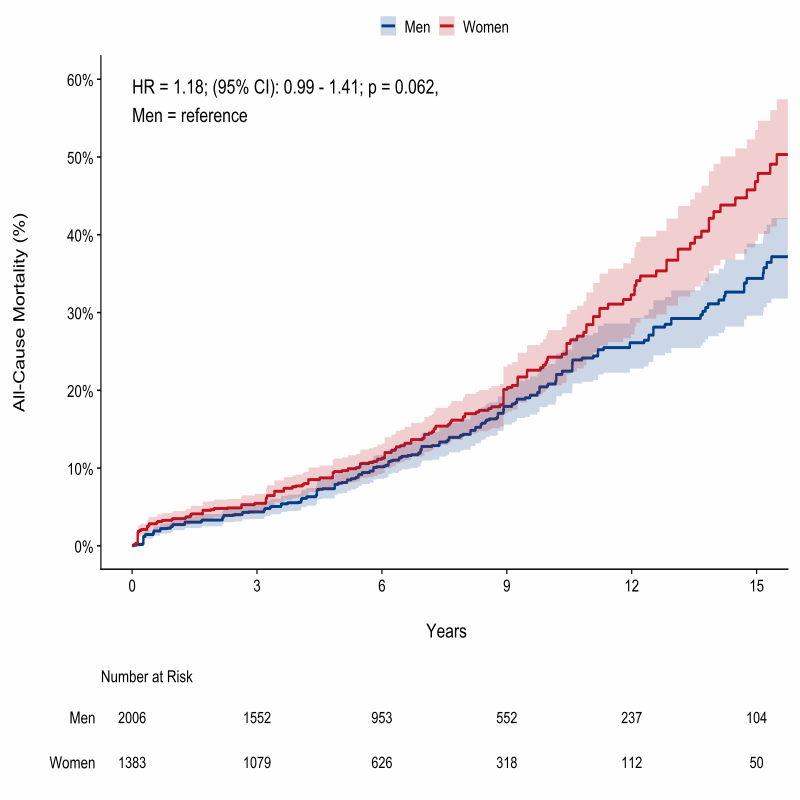


**Supplementary Figure S14**: Subgroup analysis of long-term mortality in patients undergoing mitral valve surgery for degenerative mitral regurgitation. A strong but statistically non-significant trend was observed towards worse mortality in women.


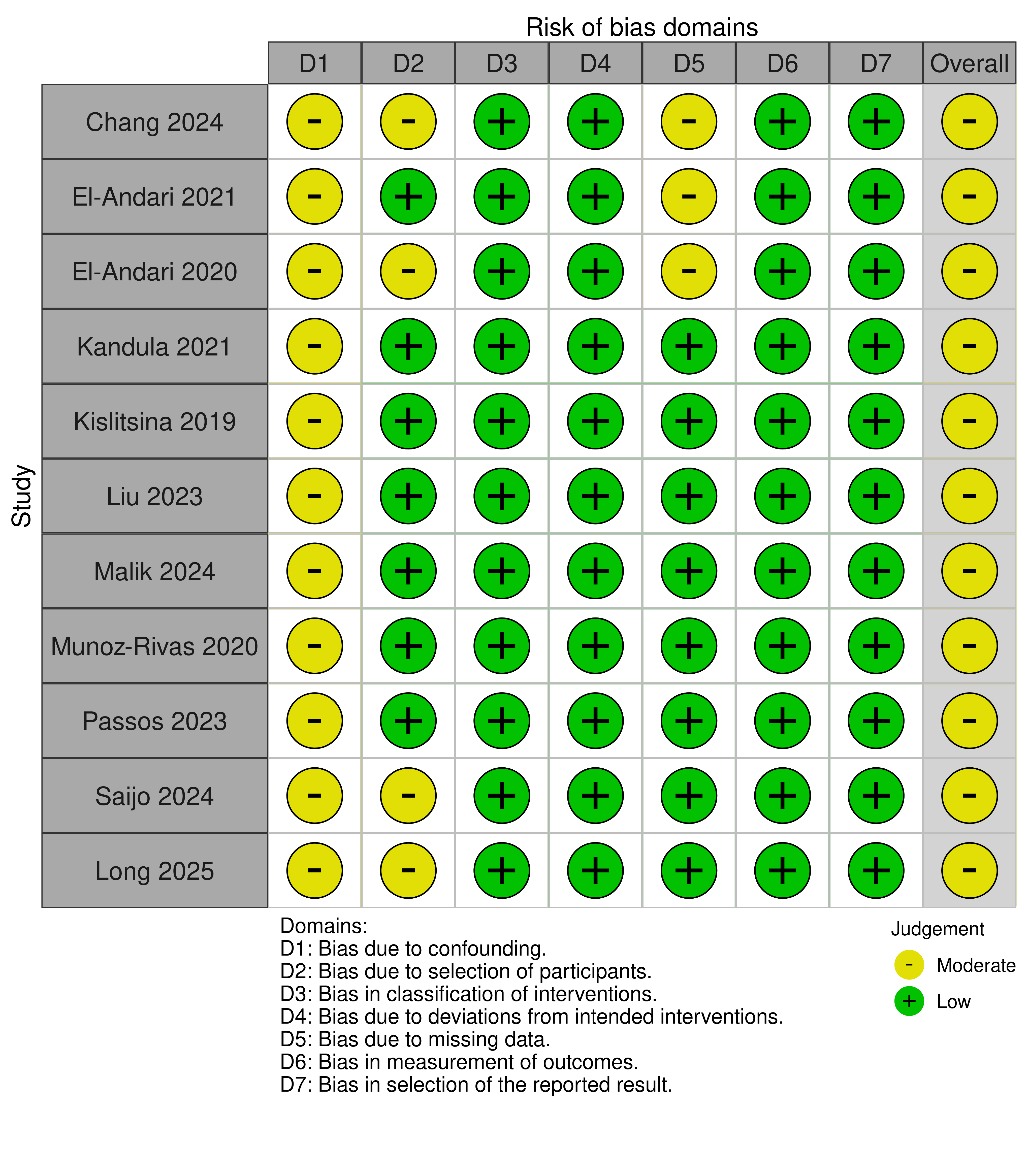


**Supplementary Figure S15**: Traffic light plot for risk of bias in observational studies. All studies were rated as being at moderate risk of bias due to either residual confounding, selection bias, or bias due to missing data.
